# Hybrid light-emitting diodes from anthracene-contained polymer and CdSe/ZnS core/shell quantum dots

**DOI:** 10.1186/1556-276X-9-611

**Published:** 2014-11-12

**Authors:** Ming-Lung Tu, Yan-Kuin Su, Ruei-Tang Chen

**Affiliations:** 1 Department of Electronic Engineering, Fortune Institute of Technology, Kaoshiung City 83160, Taiwan; 2 Institute of Microelectronics and Department of Electrical Engineering, National Cheng Kung University, Tainan City 70101, Taiwan; 3 Department of Electro-Optical Engineering, Southern Taiwan University of Science and Technology, Tainan City 710, Taiwan

**Keywords:** Light-emitting diode, Efficiency, Optical polymer

## Abstract

In this paper, we added CdSe/ZnS core/shell quantum dots (QDs) into anthracene-contained polymer. The photoluminescent (PL) characteristic of polymer/QD composite film could identify the energy transitions of anthracene-contained polymer and QDs. Furthermore, the electroluminescent (EL) characteristic of hybrid LED also identifies emission peaks of blue polymer and QDs. The maximum luminescence of the device is 970 cd/m^2^ with 9.1 wt.% QD hybrid emitter. The maximum luminous efficiency is 2.08 cd/A for the same device.

## Background

Poly(*p*-phenyene vinylene) (PPV) for application in optoelectronic fields had attracted great interest [1]. The polymer has certain advantages, such like low-cost, easy procession, and large-area display over light-emitting diodes (LEDs) made from inorganic material, especially in flexible displays [2]. Somehow, polymer LED (PLED) has one characteristic that its electron-injection is more difficult than its hole-injection due to the high energy barrier for electron-injection and low electron mobility in organic polymers. Therefore, one of the most important challenges in PLEDs is to balance the charge carrier injection that is essential for high efficiency. One method of adding nanoparticles into emissive material of PLED could be a good solution. Some former study used ZnO nanoparticles to enhance the electroluminescent characteristics of green polymer LED [3], or used ZnO nanorods to improve violet electroluminescence of polymer LED [4]. Moreover, the stable white light electroluminescence could be obtained from flexibly polymer/ZnO nanorods hybrid heterojunction [5].

Recently, several kinds of II-VI and III-V group quantum dots (QDs) were reported for different applications, including bio-sensing, marking and security, energy saving, and light-emitting [6-9]. The quasi-bound theory was utilized to predict about photogeneration efficiency improvement on polymer-embedded nanoparticles [10]. A hybrid light-emitting diode which consisted of polymer as well as QDs blending as the emissive layer is proposed for possible optoelectronic device. Following the combination of easy processing and flexibility of polymers and exotic optical properties of QDs, the so-called polymer-quantum-dot light-emitting diodes (PQD-LEDs) with different polymer and inorganic QD materials could be a successful candidate for visible displays [11,12].

We had reported blue electroluminescence from organic light-emitting diode with new anthracene-contained polymer. The polymer was synthesized through Suzuki coupling reaction. The electron-deficient oxadiazole and electron-rich carbazole derivatives were incorporated into the polymer for enhancing charge injection and transport. The good efficiency of LED based on anthracene-contained polymer was proved in the last study [13]. For researching in solid-state-lighting application, we add inorganic CdSe/ZnS quantum dots into anthracene-contained polymer for fabricating hybrid LED. The LEDs of single hybrid emissive layer have been fabricated and characterized in this study.

## Methods

**Figure 1:**
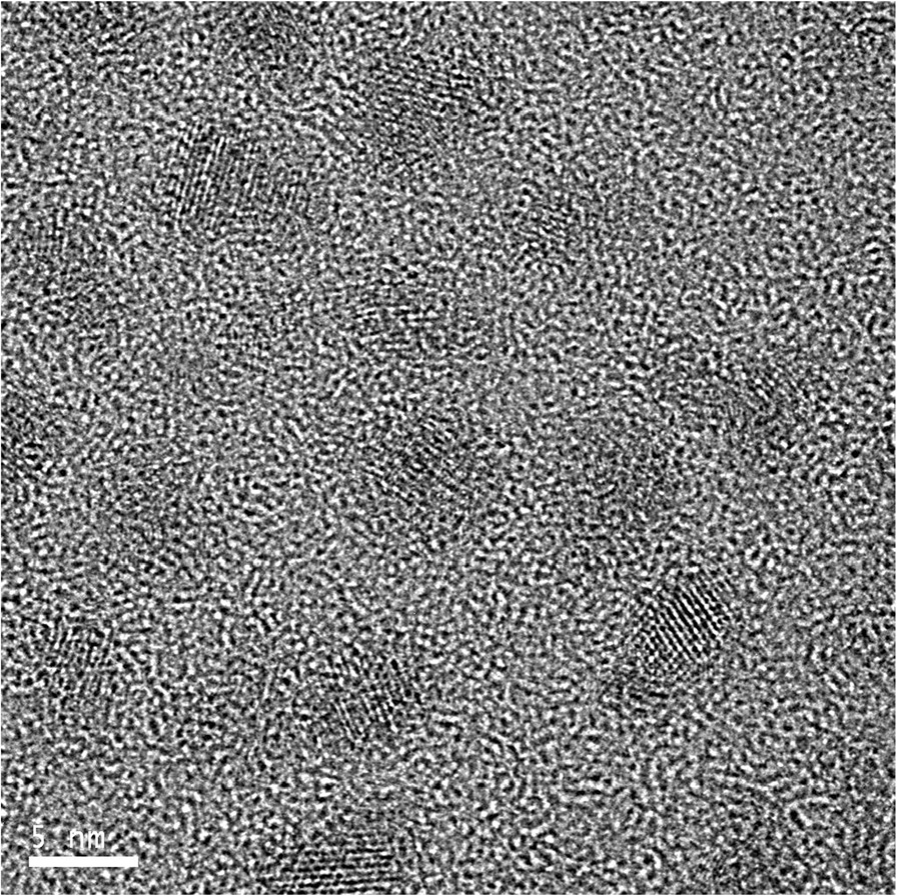
Transmission electron microscope (TEM) picture of CdSe/ZnS quantum dots.

**Figure 2:**
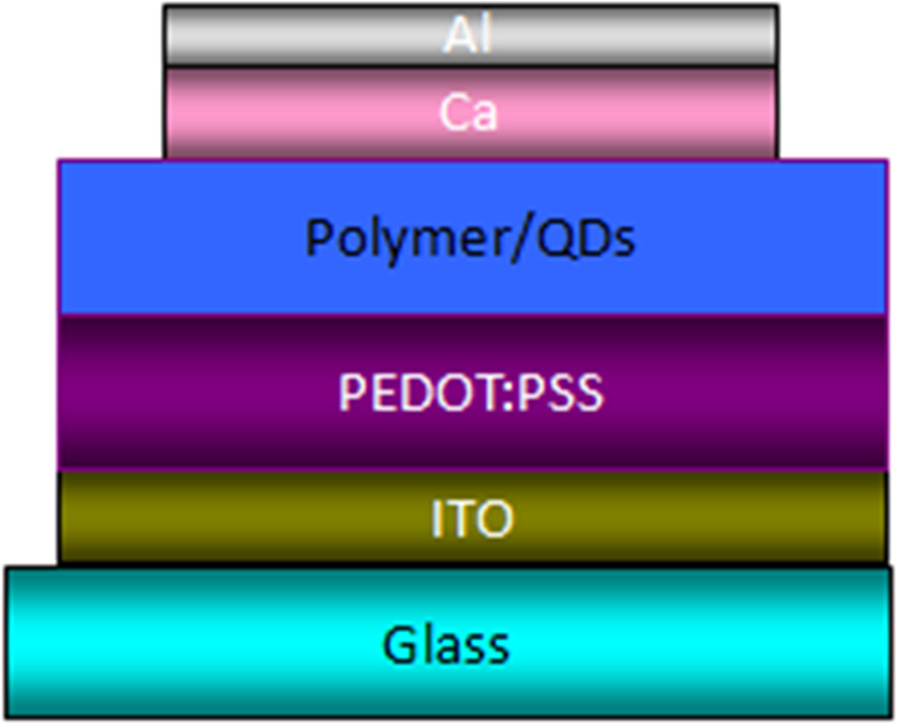
The structure of hybrid light-emitting diode.

The CdSe/ZnS QDs used in this study, which had an estimated diameter of 5.2 nm. The picture of transmission electron microscope (TEM) for QDs is shown in Figure 1. The core/shell QDs were well-dispersed in toluene with a concentration of 10 mg/ml. The polymer powder was solved into toluene for preparing solution of 3 wt.% concentration. The polymer solution and QD solution were mixed together by the 9.1 wt.% QD ratio. We had tried other QD ratios. The luminance of higher ratios LEDs could not be measured. The LED consists of composite emissive film cast from mixed solution sandwiched between a cathode and an anode. The spin speeds of cast films were 2,000, 3,000, and 4,000 r/min, from which the composited LEDs were denoted as devices 1, 2, and 3. The thicknesses of composite films for these three devices were 573, 374, and 314 nm, respectively. The full device structure is shown in Figure 2. ITO film was used for anode. The 50-nm-thick poly(3,4-ethylenedioxythiophene)-poly(4-styrene sulfonate) (PEDOT:PSS) film was for hole transport purpose. Ca/Al was deposited as the cathode by thermal evaporation. The Ca/Al films utilized for cathode were 60/120-nm-thick.

## Results and discussions

**Figure 3:**
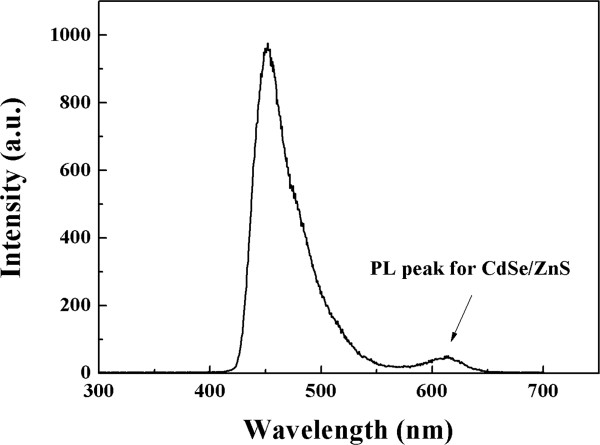
The photoluminescence spectrum of anthracene-contained blue polymer and CdSe/ZnS quantum dots composite film.

The photoluminescence (PL) measurement could be used to identify energy transition of optoelectronic semiconductor [14,15]. The PL spectrum of polymer/QD composite film is shown in Figure 3.

There are two separate PL peaks. That is two energy transitions existing in polymer/QD composite film. The first energy transition is at 452-nm wavelength and lies in the region of pure blue emission. This energy transition is owing to blue anthracene-contained polymer. This blue PL peak has 42-nm full width half maximum (FWHM). The sharp FWHM means the polymer is a very high homogeneous structure. It is obviously that QDs have energy transition at 614 nm which lies in red emission region. The PL peak of QDs has 40-nm FWHM.

**Figure 4:**
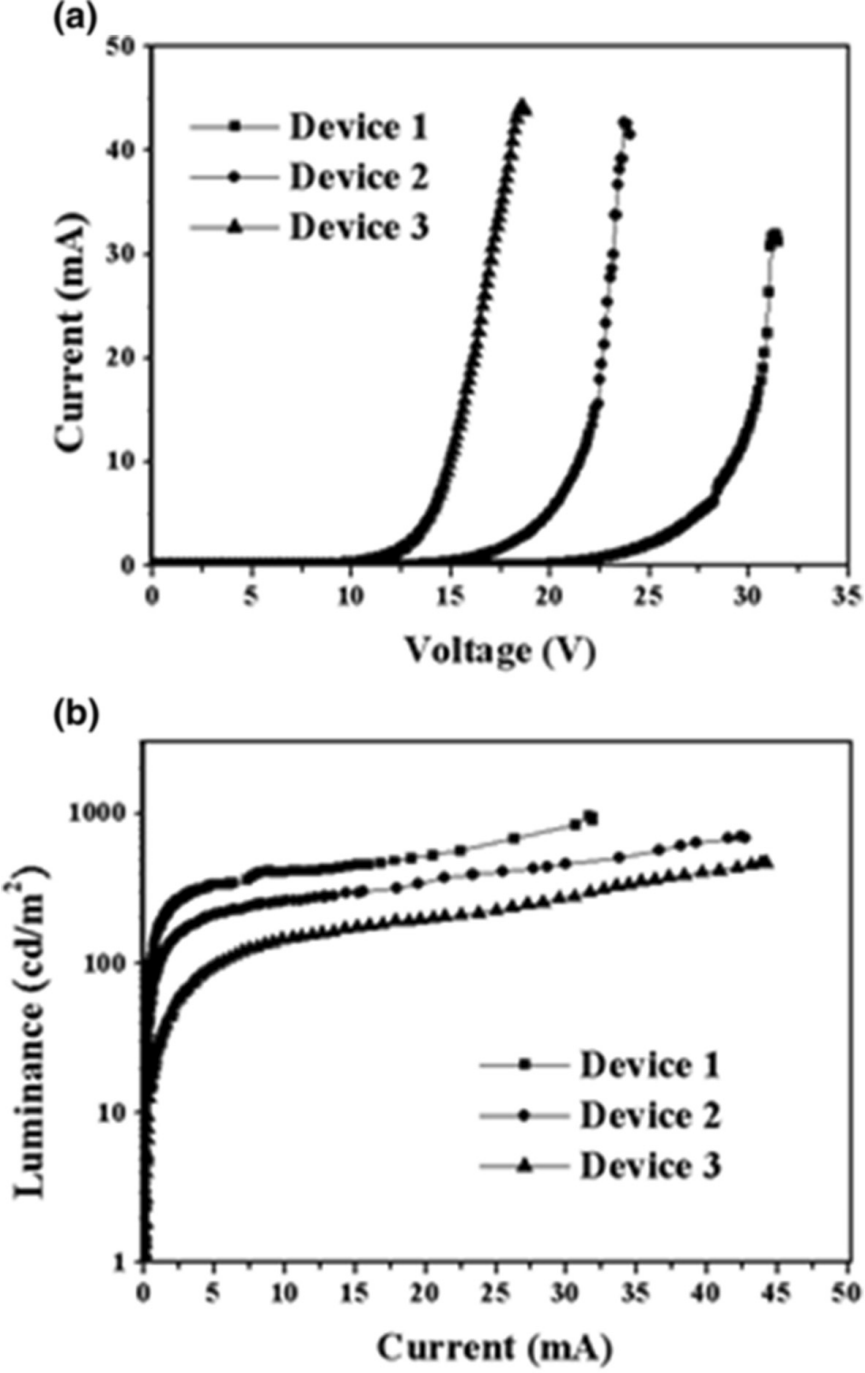
**The current-voltage ( *I *- *V *) (a) and luminance-current ( *L *- *I *) (b) characteristics of hybrid light-emitting diode.**

The current-voltage (*I*-*V*) and luminance-current (*L*-*I*) characteristics are shown in Figure 4.

It should be noticed that the devices were first biased under a moderate range to prevent the luminance degradation and voltage drift caused by overstress [16]. The threshold voltages are 26, 18, and 13 for devices, 1, 2, and 3, respectively. The thicknesses (*T*_EMI_) of polymer/QD films with spin speeds of 2,000, 3,000 and 4,000 r/min were as 573, 374, and 314 nm measured by α-step method, respectively. The polymer/QD film is thicker than pure polymer film in the same spin speed. It can be attributed to the contribution of QDs on viscosity. The threshold voltage is obviously decreased with *T*_EMI_ decreasing. If the threshold voltage divided by *T*_EMI_, about 0.41 ~ 0.45 × 10^8^ V/m would be derived. Such a high field could generate injecting of carriers into the quantum dots [17]. This field is often observed in PLEDs, and it suggests that the injection current may be limited by space charge effect or tunneling effect within polymer LED [18]. Moreover, the output luminance is also dramatically increased with *T*_EMI_ increasing as seen in Figure 4b. The PLEDs have a feature of luminance characteristics. The maximum luminance can be obtained at some point of supplied current. Luminance is gradually extinct beyond that point of supplied current. The maximum luminances are 959, 705, and 472 cd/m^2^ at supplied current of 31.6, 42.5, and 44.2 mA for devices 1, 2 and 3, respectively.

**Figure 5:**
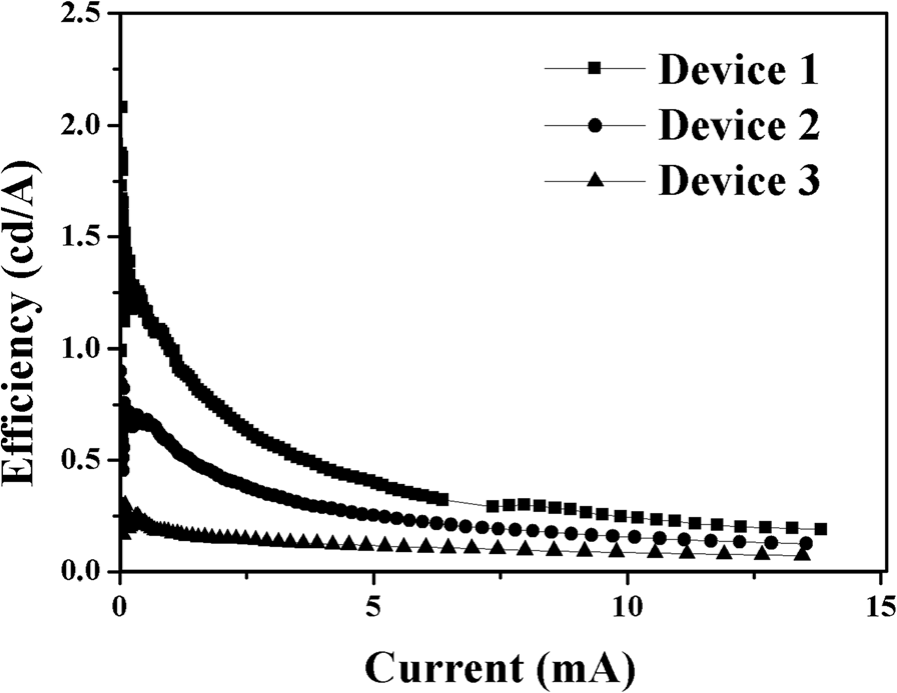
The luminous efficiency-current characteristics of hybrid light-emitting diode.

**Figure 6:**
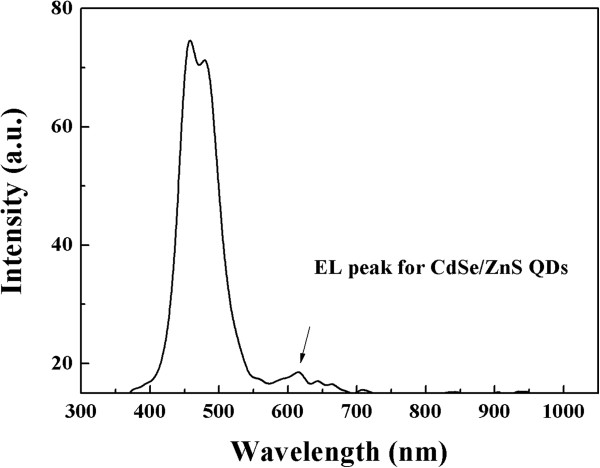
The electroluminescent spectrum of hybrid light-emitting diode.

The characteristics of luminous efficiency vs. current are shown in Figure 5. It is clearly that the efficiency is gradually decreasing beyond some maximum value even though the current injection is increasingly more. Because the polymer/QD layer is a semiconducting material, the excessive current injection turns to heat and damages the device. The maximum luminous efficiencies are 2.08, 1.25, and 0.3 cd/A for device 1, 2, and 3, respectively. The currents for maximum efficiency are 0.044, 0.018, and 0.099 mA for device 1, 2 and 3, respectively. As described before, the thicknesses of composite films for these three devices were 573, 374, and 314 nm, respectively. The electrical fields on maximum efficiency are 0.0293, 0.0291, and 0.031 V/nm for device 1, 2, and 3, respectively. The luminous efficiencies are changed with varied electrical field. The photo-generation efficiencies of the three LEDs exhibit a strong filed intensity dependency based upon quasi-bound state (QBS) theory [10].Figure 6 shows the electroluminescent (EL) spectrum of anthracene-contained blue polymer/QD hybrid LED.

In the spectrum, three definite EL peaks can be distinguished. The peaks appear at wavelengths of 458.2, 479.3, and 615 nm. The major EL peaks, 458.2 and 479.3 nm, are attributed to electroluminescence of anthracene-contained polymer [13]. These both EL peaks belong to blue emission zone. Intensity ratio of the third peak, 615 nm, compared with major 458.2 nm is 0.25. And clearly, the third EL peak could be attributed to QD electroluminescence. The luminescence of hybrid LED is mixed by blue (458.2 and 479.3-nm peaks) and red (615-nm peak) light.

**Figure 7:**
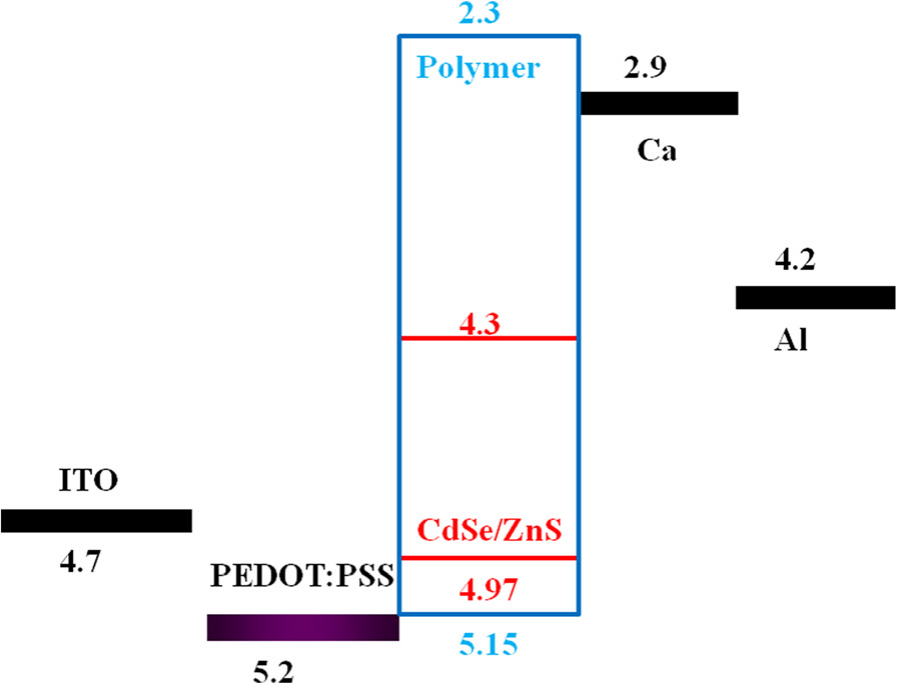
The band-diagram of hybrid light-emitting diode.

The band-diagram of hybrid LED is shown in Figure 7[19]. Blue chart is for polymer, and red chart is for QDs. As seen in Figure 7, the carriers are injected to polymer/QD layer. Some carriers cause luminescence of polymer and emit blue light. Some carriers cause luminescence of QDs and emit red light. The luminous efficiencies of hybrid LEDs decrease as compared with pure polymer LED. Even though, the EL of hybrid LED combines blue and red emissions and could have possibility for using solid-state-lighting application.

## Conclusions

In summary, the efficient hybrid LED by using anthracene-contained blue polymer and QD composite emissive film has been demonstrated. The 959 cd/m^2^ of maximum luminance is obtained at supplied 31.6 mA. The maximum luminous efficiency is 2.08 cd/A at 0.044 mA of applied current. In the EL spectrum, three definite emissions can be distinguished. The peaks appear at wavelengths of 458.2, 479.3, and 615 nm. All these three EL peaks are attributed to emit from anthracene-contained blue polymer and QDs. The hybrid LED could have possibility for using solid-state-lighting application.

## Competing interests

The authors declare that they have no competing interests.

## Authors’ contributions

M-L-T conceived the study, fabricated some of the samples, and prepared the manuscript draft and analysis. Y-K-S developed a model to describe the experimental results and helped to draft the manuscript. R-T-C helped polymer synthesis and did the discussion of the results. All authors read and approved the final manuscript.

## References

[B1] Burroughes H Bradley DDC Brown AR Marks RN Mackay K Friend RH Burns PL Holmes AB Light-emitting diodes based on conjugated polymers Nature (London) 1990 347 539 541 10.1038/347539a0

[B2] Tu M-L Su Y-K Lu W-C Yang H Kuo T-F Wen T-C Effect of post annealing on performance of polymer light-emitting devices Jap J Appl Phys 2005 44 7482 7484 10.1143/JJAP.44.7482

[B3] Rao MVM Su Y-K Huang T-S Yeh C-H Tu M-L Electroluminescent characteristics of DBPPV-ZnO nanocomposite polymer light emitting devices Nanoscale Res Let 2009 4 485 490 10.1007/s11671-009-9261-6 20596281 PMC2894341

[B4] Tu M-L Su Y-K Wu S-S Kuo T-F Wen T-C Huang C-Y Violet electroluminescence from poly(N-vinylcarbazole)/ZnO-nanorod composite polymer light-emitting devices Synth Metals 2011 161 450 454 10.1016/j.synthmet.2010.12.027

[B5] Zainelabdin A Zaman S Amin G Nur O Willander M Stable white light electroluminescence from highly flexible polymer/ZnO nanorods hybrid heterojunction grown at 50°C Nanoscale Res Let 2010 5 1442 1448 10.1007/s11671-010-9659-1 20730076 PMC2920425

[B6] Medintz IL Uyeda HT Goldman ER Mattoussi H Quantum dot bioconjugates for imaging, labeling and sensing Nat Mater 2005 4 435 446 10.1038/nmat1390 15928695

[B7] Cui D Xu J Xu SY Paradee G Lewis BA Gerhold MD Infrared photodiode based on colloidal PbSe nanocrystal quantum dots IEEE Trans Nanotech 2006 5 362 366

[B8] Likovich EM Jaramillo R Russell KJ Ramanathan S Narayanamurti V High-current-density monolayer CdSe/ZnS quantum dot light-emitting devices with oxide electrodes Adv 2011 23 4521 4525 10.1002/adma.201101782 21901762

[B9] Yu HJ Park K Chung W Kim J Kim SH White light emission from blue InGaN LED precoated with conjugated copolymer/quantum dots as hybrid phosphor Synth Metals 2009 159 2474 2477 10.1016/j.synthmet.2009.08.013

[B10] Binks DJ Quasi-bound state theory of field-dependent photogeneration from polymer-embedded nanoparticles IEEE J Quan Elec 2004 40 1140 1149

[B11] Chen HS Yeh DM Lu CF Huang CF Shiao WY Huang JJ Yang CC Liu IS Su WF White light generation with CdSe–ZnS nanocrystals coated on an InGaN–GaN quantum-well blue/green two-wavelength light-emitting diode IEEE Photon Technol Lett 2006 18 1430 1432

[B12] Mattoussi H Radzilowski LH Dabbousi BO Thomas EL Bawendi MG Rubner MF Electroluminescence from heterostructures of poly(phenylene vinylene) and inorganic CdSe nanocrystals J Appl Phys 1998 83 7965 7967 10.1063/1.367978

[B13] Tu M-L Su Y-K Wu S-S Chen R-T Electroluminescence at pure blue region from a new anthracene-contained polymer Synth Metals 2013 175 134 137

[B14] Sherriff RE Reynolds DC Look DC Jogai B Hoelscher JE Collins TC Cantwell G Harsch WC Photoluminescence measurements from the two polar faces of ZnO J Appl Phys 2000 88 3454 3457 10.1063/1.1288159

[B15] Tu ML Su YK Ma CY Nitrogen-doped *p*-type ZnO films prepared from nitrogen gas radiofrequency magnetron sputtering J Appl Phys 2006 100 053705-1 053705-3

[B16] Silvestre GCM Johnson MT Giraldo A Shannon JM Light degradation and voltage drift in polymer light-emitting diodes Appl Phys Lett 2001 50 1619 1621

[B17] Hikmet RAM Talapin DV Weller H Study of conduction mechanism and electroluminescence in CdSe/ZnS quantum dot composites J Appl Phys 2003 93 3509 3514 10.1063/1.1542940

[B18] Blom PWM de Jong MJM Electrical characterization of polymer light-emitting diodes IEEE J Sel Top Quan Elec 1998 4 105 112 10.1109/2944.669477

[B19] Mondal SP Bera S Narender G Ray SK CdSe quantum dots-poly(3-hexylthiophene) nanocomposite sensors for selective chloroform vapor detection at roocprm temperature Appl Phys Lett 2012 101 173108(1) 173108(3)

